# Effects of isoinertial or machine-based strength training on performance in tennis players

**DOI:** 10.5114/biolsport.2022.107020

**Published:** 2021-07-03

**Authors:** Jose Canós, Francisco Corbi, Joshua Colomar, Rafel Cirer-Sastre, Ernest Baiget

**Affiliations:** 1 National Institute of Physical Education of Catalonia (INEFC), University of Barcelona (UB), Barcelona, Spain; 2 National Institute of Physical Education of Catalonia (INEFC), University of Lleida (UdL), Lleida, Spain; 3 Human Movement Research Group (HMRG), University of Lleida (UdL), Lleida, Spain; 4 Sport Performance Analysis Research Group (SPARG), University of Vic – Central University of Catalonia, Barcelona, Spain

**Keywords:** Eccentric training, Racket sports, Neuromuscular performance, Physical test

## Abstract

The objective of this study was to analyze the effects of two 8-week neuromuscular training (NMT) interventions on selected physical indicators in young tennis players. Twenty-four junior male tennis players were assigned to a machine-based (MG) (n = 8), flywheel (FG) (n = 8) or a control training group (CG) (n = 8). Tests at baseline, week 4 and 8 included: countermovement jump (CMJ); speed (S; 5, 10, 15 m); agility (right [AR] and left [AL]); serve velocity (SV) and medicine ball throws (MBT; overhead [O], forehand [FH], backhand [BH]). MG and FG attained large positive effects at week 4 in CMJ, S 10 m; AR, AL and MBT FH only in FG. Regarding inter- to post-test, MG achieved large positive effects in MBT O, FH and both groups in BH. Large negative effects appeared for FG in S 5 and 10 m and AR and AL. Both NMT interventions led to positive effects from baseline to week 4 measures in CMJ, S 5 m, 10 m and agility and at week 8 in MBT. Conducting the same NMT for a longer period of time did not lead to the same improvements and other negative effects in FG appeared. Results indicate that performing these interventions with little exercise variability or load management, especially after technical-tactical sessions, could interpose further beneficial outcomes and initial gains could be impaired.

## INTRODUCTION

Competitive tennis is becoming increasingly dynamic, demanding faster game actions with more powerful skills involved [1]. Although strength and conditioning programs used to typically emphasize predominantly concentric exercises [2], most specific tennis actions imply high joint accelerations and decelerations that require concentric as well as eccentric changes in muscle length [3, 4]. In this line, during strokes, transition from pre-stretching situations to high velocity contractions appear clearly. During the preparation phase of the serve, shoulder internal rotation muscles manage high eccentric loads as do the rotator cuff, the core and lower body structures during the follow-through phase [5]. On the other hand, previous studies have observed as many as 4 changes of direction (COD) per point [6] and the number of decelerations in 3-set matches was higher than the number of accelerations [1], highlighting the relevance of eccentric actions during competition. Thus, most determinant tennis actions imply the succession of eccentric contractions just before the concentric phase, taking advantage of the elastic rebound tendency of muscle tissue in what is known as the stretch-shortening cycle (SSC) [7]. In this regard, many of the specific actions involve one or more SCC, and therefore require high intensity eccentric contractions [4, 8, 9] that must be considered when designing specific strength and conditioning programs [10].

While conventional strength and power training programs have typically involved little eccentric overload besides that which produced by the athletes own body weight and gravitational forces, rotating flywheel devices allow buildup of force throughout the concentric motion and significantly overload the subsequent eccentric phase [11]. Although it remains unclear if flywheel training is, in terms of strength indicators, superior to gravitational based programs [12], predominantly eccentric resistance training has been suggested to elicit significant gains in strength, muscle mass and power [13], helping to avoid the appearance of injuries [14]. Added to this, sport specific performance indicators seem thoroughly improved by these approaches to training [14, 15]. Also, some investigations have concluded greater gains in functional performance variables such as jump ability or speed in training groups using flywheel-based programs compared to traditional procedures [14, 16].

In this regard, trials involving the comparison of these training methods in tennis players are scarce. Although some studies show interesting results when performing plyometric training in improving performance variables such as the serve [17], some trials tested the effectiveness of functional (strength, balance, flexibility and coordination required in specific sport movements) compared to traditional (increasing strength and durability of certain muscle groups) methods on performance variables [18]. In any case, no studies have compared a flywheel-based program and a machine-based methodology. To the best of our knowledge just one study evaluated specifically the effects of predominantly eccentric training in comparison to traditional or machine-based programs in tennis players. Mont et al. [19] compared the effects of 18 sessions of isokinetic concentric versus eccentric training and observed a greater increase in average serve velocity in the eccentric group compared to the concentric group. For these reasons, it would seem logical to incorporate eccentrically biased exercises using rotational flywheel devices and tasks within a tennis strength training program. Therefore, this study aimed to evaluate the differences in tennis performance indicators after performing either a machine-based or eccentric-overloaded strength training program in young tennis players. We hypothesized a flywheel-based program would achieve greater improvements than a traditional exercise selection, as the contraction regime seen in determinant tennis actions seem to rely highly on the stretch-shortening cycle, and this seems to be further achieved when using isoinertial devices [11].

## MATERIALS AND METHODS

### Participants

Twenty-four competitive male tennis players voluntarily participated in this study (Table 1). 88% of those players were right-handed. Inclusion criteria included having more than seven years of experience playing tennis, training tennis exclusively and not having suffered any serious injury during the twelve months prior to the experiment. In addition, although players performed a fitness program based on body weight exercises, elastic tubing shoulder prevention protocols and some aerobic endurance for three days a week before taking part in the study, exclusion criteria included not having partook in any specific NMT with weight stacks or eccentric overloads within the last six months before the intervention. Moreover, two weeks prior the intervention, players ceased all physical fitness activity besides the familiarization sessions. All participants or their legal guardians agreed to and signed a formal consent form prior to the start of the program. The study was performed in accordance with current ethical standards, established in the Declaration of Helsinki of the AMM (2013) and granted approval by the relevant Institutional Ethics Committee.

**TABLE I:** Participant characteristics.

	CG (n = 8)	MG (n = 8)	FG (n = 8)	Difference (p-value)
Age (years)	15.9 ± 1.0	15.5 ± 1.2	15.5 ± 1.2	0.749
Height (cm)	174.2 ± 7.0	173.8 ± 9.6	174.9 ± 5.5	0.958
Body mass (kg)	61.4 ± 9.5	62.8 ± 10.7	66.2 ± 5.1	0.546
Training volume (h·week^-1^)	8.6 ± 0.2	9.0 ± 0.0	9.4 ± 1.1	0.079
Training background (years)	8.8 ± 1.7	9.4 ± 1.5	9.3 ± 1.7	0.719

Values are mean ± SD and p-value of the differences. CG = control group; MG = machine-based group; FG = flywheel training group.

### Experimental Design

Participants were randomly allocated into three different groups using stratified block randomization. Two of the groups were intervention groups: machine-based training group (MG; n = 8) performing machine-based neuromuscular training (MNMT) and flywheel training group (FG; n = 8) performing flywheel neuromuscular training (FNMT), and one was the control group (CG; n = 8). Only those players who participated in ≥ 80% of the NMT and technical-tactical training were included. During the study, a total of four participants (two in each of the intervention groups) were not able to complete ≥ 80% of the sessions due to circumstances unrelated to the program. As a result of these dropouts, the groups sizes were n = 8 for all groups. Both, MG and FG, completed their planned physical program after tennis sessions due to the restrictions placed on the timetable by the tennis academy. Prior to the start of the program, all participants were required to perform familiarization tests and sessions. These took place over a period of 4 weeks, with two sessions per week aiming to prepare participants for the main training program. At the end of this preparation period, all participants were instructed to rest for a period of four days. Afterwards, all subjects completed the pre-tests. Upon completion, players were randomly allocated into the three groups in order to begin the planned 16 training sessions. Inter- (at week 4) and post (at week 8) physical tests were performed again with all participants following the same conditions as pre-tests. There were no significant differences in the biometric, training characteristics (Table 1) and performance measurements before the training intervention, only between CG and FG in agility 5-0-5 left. All results were collected on an outdoor synthetic court by the same researchers. In addition to the experimental training sessions, all participants took part in a thirty-minute injury-prevention session, which included exercises working the core, shoulder rotators, stabilization, mobility and flexibility. These sessions occurred throughout the eight-week period, twice a week (Tuesdays and Thursdays). Also, all players participated in similar two-hour long tennis (i.e., on-court technical and tactical skills) training sessions three times a week (Monday, Wednesday and Friday), resulting in a total of six hours per week. Throughout the study, players were not allowed to modify the style or technique of their strokes, nor the string tension and racket they used. During the course of the intervention no training-related injuries occurred.

### Measurements

All measurements used have been previously recommended to assess performance variables in tennis players [20] and showed good-toexcellent test-retest reliability (ICC = 0.85 to 0.99). For data analysis, only the best result of attempts was selected and recorded. The order of the tests was randomized to isolate the possible influence of fatigue.

### Countermovement jump (CMJ)

Vertical jump was measured without the use of arm swing and was executed on a contact platform (Chronojump-Boscosystem, Barcelona, Spain), attached to a micro-controller Chronopic, and to a specific software (Chronojump). Each player performed 3 attempts with 1 minute of passive recovery between each repetition.

### Speed (5, 10 and 15 m)

Linear average speed was measured using sprint tests with distances of 5, 10 and 15 m. Players were given three attempts for each distance and results were measured using photoelectric cells (Artek PNP response time < 0.5 ms at 1 kHz, Barcelona, Spain). All participants initiated their run from 50 cm behind the photocell gate using the same chosen standing position, legs parallel to one another. 2 minutes of passive rest were given between attempts.

### Agility (modified 5-0-5 agility right and left test)

Each participant was allowed to select their own starting position as long as their preferred foot was behind the line. All players then accelerated forward at their maximal speed and were required to pivot on one leg, rapidly changing direction 180° and then returning to the start line as quickly as possible. Participants were required to perform the test two times pivoting on the left foot and twice on the right foot. Between each test, there was a rest of 2 minutes. Results were registered using the same timing gates as before.

### Serve velocity (SV)

Following similar protocols [8, 21], SV was measured using a radar gun (Stalker Professional Sports Radar, Plymouth, MN, USA), which was positioned three meters behind the server, at the centre of the baseline and aimed at the approximate point of contact with the ball during the serve. Participants served at a maximum speed five times from each side of the court using new balls (HeadTour^®^) with a rest of 25 seconds between each serve. Only each player’s fastest serve from each side that landed within the respective service box were recorded.

### Medicine ball throws (MBT)

Players completed MBT using overhead, forehand and backhand movements. All throws were completed using a 3-kg medicine ball. For the overhead throws, players were instructed to use an open stance position. While performing the forehand and backhand throws, players used a closed stance. During all throws, subjects were allowed to flex and bend their legs but were obliged to maintain contact with the ground at all times. A radar gun was used to measure the speed of each attempt. Players were given two attempts for each position and were allowed 30 seconds rest between throws.

### Training intervention

**FIG. 1:**
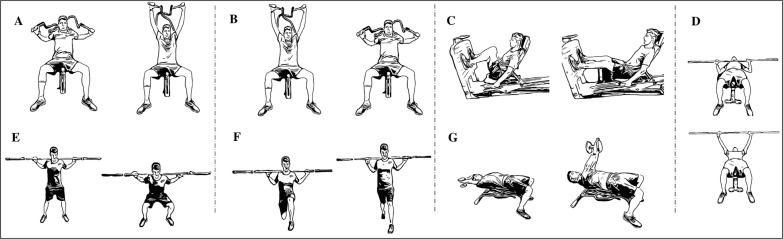
Machine-based neuromuscular training (MNMT) program intervention. A = shoulder press machine; B = lateral pulldown machine; C = complete leg press machine; D = bench press on multipower; E = half squat on multipower; F = forward lunge on multipower; G = dumbbell pullover.

**FIG. 2:**
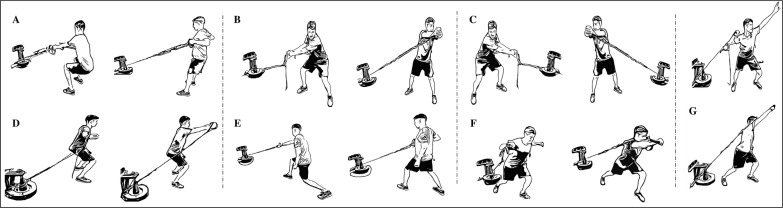
Flywheel-based neuromuscular training (FNMT) program intervention. A = low row 90º; B = forehand closed stance; C = backhand closed stance; D = one handed chest crossover; E = one-handed low row with one step; F = global chest press with one step; G = one-handed shoulder press.

Two days per week (Monday and Wednesday) subjects performed a MNMT (Table 2A) or a FNMT (Table 2B) program consisting of 3 sets of 5–6 exercises (Figures 1 and 2) of 6–8 repetitions followed by a block of specific exercises (SE) (2–3 sets of 5–6 exercises) at the end of the session, following organization and loads summarized in Table 2. Following literature, in order to achieve a significant eccentric overload during the eccentric phase of contraction, participants were asked to control the execution technique and delay the braking action in the eccentric phase when executing flywheel exercises [22]. In order to even intensity and loads as much as possible between methodologies, following training sessions, each participant was asked to complete a self-assessment using modified Börg’s rate of perceived exertion (RPE) [23], resulting in a mean value throughout the 8 weeks of training of 6.2 ± 0.6 for MNMT and 6.1 ± 0.7 for FNMT.

**TABLE 2:** Machine-based (A) and flywheel (B) neuromuscular training interventions programs.

A - Machine-based neuromuscular training (MNMT)										
Week	Sessions per week (no.)	Exercises (no.)	Sets (no.)	Reps (no.)	1RM (%)	Intended Velocity	Mean RPE	Rest set/round (min)	Exercise Program	Specific Exercises (SE)
1–2	2	5	3	8	50–60	Max	5.7 ± 0.8	1.5/3	A) Shoulder press machine; B) Lateral pulldown machine; C) Complete leg press machine; D) Bench press on multipower; E) Half squat on multipower	2 sets of: A) 5 m sprint; B) 10 m sprint; C) 15 m sprint; D) 505-agility right; E) 505-agility left; F) 6 reps of CMJ
3–4	2	5	3	8	50–60	Max	6.4 ± 0.6	1.5/3		
5–6	2	5	3	6	60–70	Max	6.5 ± 0.6	1.5/3	A) Shoulder press machine; B) Lateral pulldown machine; C) Complete leg press machine; F) Forward lunge; G) Dumbbell pullover	3 sets of: A) 5 m sprint; B) 10 m sprint; C) 15 m sprint; D) 505-agility right; E) 505-agility left; F) 6 reps of CMJ; G) MBT; H) 6 reps of 2-handed of 3 kg MBT (forehand, backhand and overhead)
7–8	2	5	3	6	60–70	Max	6.1 ± 0.5	1.5/3		

**B - Flywheel neuromuscular training (FNMT)**										
**Week**	**Sessions per week (no.)**	**Exercises (no.)**	**Sets (no.)**	**Reps (no.)**	**Overloads (no.)**	**Intended Velocity concentric phase**	**Mean RPE**	**Rest set/ round (min)**	**Exercise Program**	**Specific Exercises (SE)**

1–2	2	4	3	8	1	Max	5.7 ± 0.9	1.5/3	A) Low row 90º; B) Forehand closed stance; C) Backhand closed stance; D) One-handed chest crossover	2 sets of: A) 5 m sprint; B) 10 m sprint; C) 15 m sprint; D) 505-agility right; E) 505-agility left; F) 6 reps of CMJ.
3–4	2	4	3	8	1	Max	6.3 ± 0.5	1.5/3		
5–6	2	5	3	6	2	Max	6.3 ± 0.9	1.5/3	E) One handed low row with one step; B) Forehand closed stance; C) Backhand close stance; F) Global chest press with one step; G) One-handed shoulder press	3 sets of: A) 5 m sprint; B) 10 m sprint; C) 15 m sprint; D) 505-agility right; E) 505-agility left; F) 6 reps of CMJ; G) 6 reps of two-handed of 3 kg MBT (forehand, backhand and overhead)
7–8	2	5	3	6	2	Max	6.1 ± 0.5	1.5/3		

MNMT = machine-based neuromuscular training; FNMT = flywheel neuromuscular training; RPE = rate of perceived exertion; CMJ = countermovement jump; MBT = medicine ball throws.

### Statistical Analyses

Data are presented as mean ± standard deviation (SD). Normality of residuals and homogeneity of variances were assessed using Shapiro-Wilk and Levene tests, respectively. The effects of the intervention on each outcome variable were evaluated using linear mixed-effects models [24]. As fixed effects, we entered group and time with interaction term into the model. As random effects, we added random intercepts at participant level, accounting for correlated measurements from the same participant and individual variation at baseline. Reference levels for contrasts were set to time = baseline and group = control. Pairwise comparisons between moments for each group and between groups at each moment were made using estimated marginal means, using the Kenward-Roger’s method to estimate confidence intervals, and controlling for multiple comparisons using Tukey’s adjustment [25]. Model estimates were reported as 95% confidence intervals (95% CI). When confidence intervals for the differences did not include 0, effect sizes (ES) were reported using Cohen’s *d*., the criteria to interpret the magnitude of the ES were 0.0–0.2 trivial, 0.2–0.6 small, 0.6–1.2 moderate, 1.2–2.0 large, and > 2.0 very large [26]. Statistical analyses were made using R v4.0.1 (The R Foundation for Statistical Computing, Vienna, Austria), utilizing the packages *lme4* (v1.1–23), *lmerTest* (v3.1–2), *emmeans* (v1.4.8), and *effectsize* (v0.3.1).

## RESULTS

**FIG. 3:**
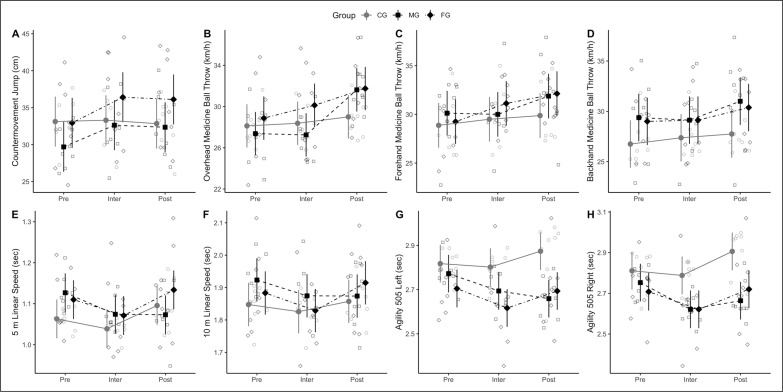
Individual points and mixed-effects models estimate for machine-based group (MG), flywheel group (FG) and control group (CG) at week 0 (Pre), week 4 (Inter) and week 8 (Post) of training intervention.

Individual points and mixed-effects models estimate have been plotted in Figure 3. Percentage and magnitude of changes are summarized in Table 3. There were main differences among groups in agility (left, *p* = 0.019; right, *p* = 0.027), but not in any other of the tested variables. Main differences over time were evident in CMJ (*p* < 0.001), agility (left, *p* = 0.003; right, *p* < 0.001), linear speed (5 m, *p* < 0.001; 10 m, *p* < 0.001; 15 m, *p* = 0.016), and MBT (overhead, *p* < 0.001; forehand, *p* < 0.001; backhand, *p* < 0.001). In addition, there was group × time interaction in CMJ (*p* = 0.002), linear speed at 5 m (*p* = 0.01) and 10 m (*p* = 0.005), agility (left, *p* = 0.002; right, *p* < 0.001), and overhead MBT *(p* < 0.001).

**TABLE III:** Changes in the performance variables obtained during pre- inter- and post strength training intervention.

Jump height	CG (n = 8)				MG (n = 8)				FG (n = 8)				
	ES (95% CI)	Change (%)		Descriptor	ES (95% CI)	Change (%)		Descriptor	ES (95% CI)		Change (%)	Descriptor	
CMJ (cm) Inter – Pre Post – Pre Post – Inter	0.16 (-0.91 – 1.23) -0.28 (-1.36 – 0.79) -0.52 (-1.61 – 0.57)	0.6 -0.9 -1.5		Trivial Small Small	1.05 (-0.09 – 2.20)* 1.06 (-0.08 – 2.21)* -0.16 (-1.24 – 0.91)	9.8 9.1 -0.6		Moderate Moderate Trivial	1.34 (0.15 – 2.52)* 1.29 (0.11 – 2.47)* -0.2 (-1.28 – 0.88)	10.6 9.7 -0.8		Large Large Small	

**Speed**													
Sprint 5 m (s) Inter – Pre Post – Pre Post – Inter	-1.78 (-3.05 – -0.51) 0.71 (-0.40 – 1.81) 1.03 (-0.11 – 2.18)*	-1.9 3.8 5.8	Large Moderate Moderate		-1.58 (-2.81 – -0.35)* -0.69 (-1.79 – 0.42)* -0.02 (-1.09 – 1.05)		-5.3 -5.3 0	Large Moderate Trivial	-0.63 (-1.73 – 0.47) 0.4 (-0.68 – 1.48) 1.12 (-0.03 – 2.27)*	-3.6 1.8 5.6			Moderate Small Moderate

Sprint 10 m (s) Inter – Pre Post – Pre Post – Inter	-2.07 (-3.4 – -0.74) 0.32 (-0.76 – 1.39) 1.20 (0.03 – 2.36)	-1.1 0.5 1.6	Very large Small Large		-1.01 (-2.15 – 0.13)* -0.79 (-1.9 – 0.33)* -0.01 (-1.09 – 1.06)		-2.6 -2.6 0	Moderate Moderate Trivial	-0.79 (-1.90 – 0.32)* 0.37 (-0.71 – 1.45) 2.41 (1.0 – 3.82)*	-2.7 1.6 4.4			Moderate Small Very large

Sprint 15 m (s) Inter – Pre Post – Pre Post – Inter	-1.21 (-2.38 – -0.04) -0.10 (-1.17 – 0.97) 0.12 (-0.95 – 1.20)	-1.2 -0.8 -0.4	Large Trivial Trivial		-1.23 (-2.4 – -0.06) -0.75 (-1.86 – 0.36) 0.58 (-0.51 – 1.67)		-2.9 -1.5 0.8	Large Moderate Small	-0.61 (-1.70 – 0.49) 0.23 (-0.85 – 1.31) 1.03 (-0.11 – 2.17)	-1.6 0.8 2.4			Moderate Small Moderate

**Agility**													
5-0-5 Right (s) Inter – Pre Post – Pre Post – Inter	-1.02 (-2.16 – 0.12) 2.21 (0.85 – 3.57)* 2.57 (1.12 – 4.02)*	-0.7 3.6 4.3	Moderate Very large Very large		-2.02 (-3.34 – -0.7)* -1.04 (-2.19 – 0.10)* 0.49 (-0.60 – 1.57)		-4.7 -3.3 1.5	Very large Moderate Small	-0.86 (-1.98 – 0.26)* 0.13 (-0.95 – 1.20) 1.4 (0.20 – 2.60)*	-3.3 0.37 3.8			Moderate Trivial Large

5-0-5 Left (s) Inter – Pre Post – Pre Post – Inter	-0.61 (-1.71 – 0.49) 0.54 (-0.55 – 1.63) 0.72 (-0.39 – 1.82)	-0.7 1.8 2.5	Moderate Small Moderate		-0.88 (-2.01 – 0.24)* -1.07 (-2.21 – 0.08)* -0.34 (-1.42 – 0.74)		-2.9 -4.0 -1.1	Moderate Moderate Small	-0.84 (-1.96 – 0.27)* -0.16 (-1.24 – 0.91) 1.5 (0.28 – 2.71)*	-3.0 -0.4 2.7			Moderate Trivial Large

**SV**													
Right (km·h^-1^) Inter – Pre Post – Pre Post – Inter	0.33 (-0.75 – 1.41) -0.06 (-1.14 – 1.01) -0.54 (-1.63 – 0.56)	1.3 -0.4 -1.7	Small Trivial Small		-0.3 (-1.38 – 0.78) 0.26 (-0.82 – 1.34) 0.51 (-0.58 – 1.60)		-1.3 1.8 3.2	Small Small Small	-0.38 (-1.47 – 0.70) 0.32 (-0.76 – 1.40) 0.65 (-0.45 – 1.75)	-1.2 2.5 3.7			Small Small Moderate

Left (km·h^-1^) Inter – Pre Post – Pre Post – Inter	-0.17 (-1.24 – 0.90) -0.21 (-1.29 – 0.86) -0.04 (-1.11 – 1.03)	-1.1 -1.3 -0.2	Trivial Small Trivial		-0.13 (-1.2 – 0.95) 0.22 (-0.85 – 1.30) 0.47 (-0.61 – 1.56)		-0.5 1.1 1.5	Trivial Small Small	-0.35 (-1.43 – 0.73) -0.14 (-1.22 – 0.93) -0.01 (-1.08 – 1.06)	-1.0 -1.1 -0.1			Small Trivial Trivial

**MBT**													
Overhead (km·h^-1^) Inter – Pre Post – Pre Post – Inter	0.33 (-0.75 – 1.41) 0.83 (-0.28 – 1.95) 0.79 (-0.32 – 1.91)	1.1 3.2 2.1	Small Moderate Moderate		-0.05 (-1.12 – 1.02) 1.84 (0.56 – 3.12)* 2.34 (0.95 – 3.73)*		-0.4 15.3 15.8	Trivial Large Very large	0.79 (-0.32 – 1.91) 1.86 (0.58 – 3.15) 0.96 (-0.17 – 2.09)	4.2 10.0 5.7			Moderate Large Moderate

Forehand (km·h^-1^) Inter – Pre Post – Pre Post – Inter	1.14 (-0.01 – 2.3) 1.25 (0.08 – 2.42) 0.48 (-0.61 – 1.56)	2.1 3.5 1.4	Moderate Large Small		-0.05 (-1.12 – 1.03) 0.70 (-0.41 – 1.80)* 1.22 (0.05 – 2.38)*		-0.3 6.0 6.3	Trivial Moderate Large	0.82 (-0.30 – 1.93)* 1.57 (0.35 – 2.80)* 0.79 (-0.32 – 1.90)	6.1 9.6 3.2			Moderate Large Moderate

Backhand (km·h^-1^) Inter – Pre Post – Pre Post – Inter	1.14 (-0.01 – 2.3) 1.77 (0.50 – 3.03) 0.69 (-0.42 – 1.79)	2.2 3.7 1.5	Moderate Large Moderate		-0.33 (-1.41 – 0.75) 1.09 (-0.06 – 2.24)* 1.03 (-0.11 – 2.17)*		-1.0 5.4 6.5	Small Moderate Moderate	(-0.97 – 1.18) 0.74 (-0.37 – 1.84)* 0.85 (-0.27 – 1.97)*	0.3 4.8 4.5			Trivial Moderate Moderate

CG = control group; MG = machine-based group; FG = flywheel group; ES = effect size; CI = confidence intervals; CMJ = countermovement jump; SV = serve velocity; MBT = medicine ball throws;

* *p* < 0.05 within group differences. Magnitudes of ESs were assessed using the following criteria: < 0.2 = trivial, 0.2–0.6 = small, 0.6–1.2 = moderate, 1.2–2.0 = large, > 2.0 = very large.

CG had comparable outcomes over time in all variables, except a decrease in linear 5 m speed (95% CI = 0.01 – 0.1 s, *p* = 0.009, *d* = 0.48) and agility left (95% CI = 0 – 0.14 s, *p* = 0.048, *d* = 0.38) and right (95% CI = 0.06 – 0.18 s, *p* < 0.001, *d* = 0.75) observed at week 8. MG showed 4-week improvements in CMJ (95% CI = 1.33 – 4.53 cm, *p* < 0.001, *d* = 0.69), 5 (95% CI = -0.1 – -0.01 s, *p* = 0.016, *d* = -0.45) and 10 m (95% CI = -0.09 – -0.01 s, *p* = 0.020, *d* = -0.43) speed, as well as left (95% CI = -0.15 – 0 s, *p* = 0.027, *d* = -0.41) and right (95% CI = -0.19 – -0.07 s, *p* < 0.001, *d* = -0.83) agility. These improvements were comparable at week 8. Overhead (95% CI = 3.02 – 5.7 km·h^-1^, *p* < 0.001, *d* = 1.21), forehand (95% CI = 0.45 – 3.30 km·h^-1^, *p* = 0.007, *d* = 0.49) and backhand (95% CI = 0.86 to 2.9 km·h^-1^, *p* < 0.001, *d* = 0.69) MBT improved at week 8. FG showed 4-week improvements in CMJ (95% CI = 1.9 – 5.1 cm, *p* < 0.001, *d* = 0.81), that were maintained until week 8. 5 m speed did not change at week 4 and decreased at week 8 (95% CI = 0.02 – 0.11 s, *p* = 0.004, *d* = -0.33). Similarly, 10 m speed (95% CI = -0.1 – -0.01 s, *p* = 0.009, *d* = -0.48) as well as left (95% CI = -0.16 – -0.02 s, *p* = 0.012, *d* = -0.46) and right (95% CI = -0.15 – -0.03 s, *p* = 0.003, *d* = -0.55) agility improved at week 4, but then decreased at week 8. MBT, by contrast, improved at week 4 for forehand throws (95% CI = 0.45 – 0.54 km·h^-1^, *p* = 0.007, *d* = 0.49) which was maintained until week 8, and did not change at week 4 for overhead and backhand throws which then improved at week 8 (overhead, 95% CI = 0.27 to 3.0 km·h^-1^, *p* < 0.001, *d* = 0.45; and backhand, 95% CI = 0.23 – 2.3 km·h^-1^, *p* = 0.013, *d* = 0.46).

## DISCUSSION

Findings in this study suggest that performance indicators, specifically lower body strength, short sprinting and agility are improved in just 4 weeks of either machine or flywheel based NMT programs. Also, improvements in upper body power (i.e., MBT) could be observed after 8 weeks of intervention in both groups. Group interactions showed slightly greater results in CMJ for the FG, while 5 m, 10 m, agility and overhead MBT were further improved in the MG in comparison to the FG. On the other hand, no changes could be observed in SV or 15 m sprinting after any of the training programs. Performing these interventions for up to 8 weeks with no variations or load adjustments, especially after technical-tactical sessions, could interpose further beneficial outcomes and even lead to decreases in performance, as seen in certain variables.

To our knowledge, no previous studies have focused on comparing MNMT and FNMT in young tennis players, however, similar approaches regarding traditional strength training (i.e., free weights, machines or plyometrics) have achieved positive results regarding physical determinants [4, 9, 10, 27]. In this investigation, results indicate improvements at week 4 in 10 m sprint and 505 agility right and left in both NMT programs. Nevertheless, significant positive changes in 5 m sprint and slightly greater gains in 505 agility right (-4.7%; ES = -2.02 vs. -3.3%; ES = -0.86) could be observed in the MG, while 10 m sprints and agility 505 left achieved similar improvements in both groups. Most likely, these differences may be due to program differences and the fact that MG included two basic strength exercises involving legs (i.e., complete leg press machine and half squat on multipower), while the FG performed predominantly upper body exercises with the only lower body implication of accompanying the movement during execution. Compared to the FG, a higher volume of strength training targeted to the lower body was performed in the MG and thus, greater improvements could be explained because of this. Literature suggests the benefit of strength training (i.e., squatting based exercises) in sprinting and COD speed, especially when combined with motions that create horizontal exertion of force to provide transfer throughout the movement [28]. Following this idea, as SE were performed after every NMT session, the MG could have been benefited by the combined effect of strength training and more specific actions (i.e., contrast approach) such as sprinting and agility, as similarly seen in previous investigations that combined strength interventions and specific COD training [10]. Despite this, the FG program included exercises mainly in the horizontal plane, and we could assume it would be more plane specific to sporting actions such as sprinting and COD. Nevertheless, results here are in accordance with those reported in Hoyo et al., 2016 [29], observing similar results in vertical strength training when compared to tasks with predominantly horizontal force application.

Sprint and agility indicators stalled in the MG and even decreased in the FG from week 4 to week 8 measurements. Although from week 4 to 8 some changes were included (i.e., pullovers in MNMT, unilateral predominance in FNMT exercises) in both programs (see Table 2), and an extra set in SE, this could be insufficient to elicit further changes in specific sporting actions tested here. As seen previously [9, 11], NMT programs can increase sprint and agility performance variables in young athletes following 6 or more weeks of training, suggesting maintenance and decreases here could be caused by insufficient load variability alongside adaptation to SE. The importance of applying changes as training programs advance in duration seems clear and adjustments regarding not only volume and intensity but exercise selection seem important factors affecting training outcomes [30]. Moreover, results in previous studies registered no variations in agility and speed indicators in young tennis players when performing NMT programs after the technical and tactical sessions, as these could lead to a fatigued state and reduce the quality of subsequent NMT outcome [14]. Because of this, MG improvements could be impaired in the second part of the program (weeks 5 to 8) as training volume accumulates. Regarding the reductions in performance in FG, literature suggests a high degree of fatigue induced by eccentric training that affects force production and neural control and continues in time if an appropriate recovery window is not respected [31]. This, alongside the previous statements and the fact that these players were untrained in the use of flywheel devices could have induced results closer to detraining rather than maintenance in values.

Regarding jumping capacity, both groups acquired improvements in CMJ after 4 and 8 weeks of training with slightly greater gains in the FG. As both programs seem optimal to develop power in jumping actions, these small differences could possibly be explained by the fact that flywheel devices enhance the eccentric phase of the motion and allow the athlete take advantage of energy storage towards performance in the subsequent concentric phase. These greater results in flywheel interventions compared to traditional methodologies have previously been observed throughout literature [14].

MBT were generally unaltered during the first 4 weeks of intervention in both groups. Although these actions involve full body coordinated motions, a great deal of the action’s outcome is influenced by upper body segments [32]. In this regard, no significant changes comparing baseline and week 4 results were found except for the FG forehand MBT. In this case, it could be that the higher volume of upper body strength training the FG performed (4/4 exercises vs. 3/5 in the MG) could explain the fact that just this group achieved significant improvements in one of the MBT. Nevertheless, as the intervention program advances in duration and analyzing baseline values to week 8 results, data suggests improvements in both groups, with greater progress in the overhead MBT in the MG at week 8. Unlike the first 4 weeks of intervention where FG had a major volume of upper body strength training than MG, as the program advances in duration, increases in load and variability may become more important than total volume and MG would result benefited. Starting from week 5, the inclusion of MBT in the SE sessions could have triggered major improvements in the MG as they may have benefited from the combination of increased load in their program (50% to 70% 1RM) and the inclusion of specific transfer exercises (MBT included in SE), attaining the benefits of contrast training in upper body, throwing or striking motions [33]. In the same line, FG also increased performance in selected variables, restating the possible beneficial effect of including MBT in the SE.

Regarding SV, no significant differences were found in any of the groups throughout the intervention program, suggesting that improvements in MBT do not necessarily follow SV improvements, as they may not correlate strongly [21]. Contrary to this, studies have generally found effective the use of various training methods to increase velocity production in the tennis serve [7, 34]. As well documented in literature, the tennis serve is a complex motor skill that involves a great deal of physical aspects that influence its outcome (i.e., strength, range of movement and technique) [8, 35, 36]. Because of this, it has been suggested that training programs that more effectively simulate the motion, include the transfer of force throughout the kinetic chain and involve core stabilization of trunk and pelvic rotation are strongly recommended for tennis players [36]. In this program, only MBT included and executed during SE from weeks 5 to 8 may have been insufficient to reflect improvements in SV, whereas both MG and FG resistance training exercises did not respond to these necessities and therefore did not produce significant changes in performance.

Both NMT presented here seem in general useful tools to improve physical capacities of young tennis players in relatively short periods of time. In a sport like tennis, with little preparation periods, constant travels and limited training availability, this training approach could be useful for players and coaches. In fact, the combination of both training interventions alongside approaches that include sport-specific actions could achieve higher gains than performing them separately. Nevertheless, applying these programs with low exercise variability for long periods, especially following flywheel interventions, could impair gains and even decrease performance.

## CONCLUSIONS

In conclusion, this study shows that 4 weeks of both, traditional and flywheel based NMT achieve improvements in CMJ, sprints and agility indicators, while MBT improvements could be observed after 8 weeks of intervention. However, performing these interventions with little exercise variability or load management, especially after technical-tactical sessions, could interpose further beneficial outcomes and gains could be impaired.
